# Hyaluronidases in Human Diseases

**DOI:** 10.3390/ijms22063204

**Published:** 2021-03-22

**Authors:** Aditya Kaul, Walker D. Short, Xinyi Wang, Sundeep G. Keswani

**Affiliations:** 1 Laboratory for Regenerative Tissue Repair, Division of Pediatric Surgery, Department of Surgery, Texas Children’s Hospital, Houston, TX 77030, USA; aditya.kaul@bcm.edu (A.K.); walker.short@bcm.edu (W.D.S.); 2 Department of Surgery, Baylor College of Medicine, Houston, TX 77030, USA

**Keywords:** disease, hyaluronic acid, hyaluronidase, molecular weight

## Abstract

With the burgeoning interest in hyaluronic acid (HA) in recent years, hyaluronidases (HYALs) have come to light for their role in regulating catabolism of HA and its molecular weight (MW) distribution in various tissues. Of the six hyaluronidase-like gene sequences in the human genome, *HYALs 1* and *2* are of particular significance because they are the primary hyaluronidases active in human somatic tissue. Perhaps more importantly, for the sake of this review, they cleave anti-inflammatory and anti-fibrotic high-molecular-weight HA into pro-inflammatory and pro-fibrotic oligosaccharides. With this, HYALs regulate HA degradation and thus the development and progression of various diseases. Given the dearth of literature focusing specifically on HYALs in the past decade, this review seeks to expound their role in human diseases of the skin, heart, kidneys, and more. The review will delve into the molecular mechanisms and pathways of HYALs and discuss current and potential future therapeutic benefits of HYALs as a clinical treatment.

## 1. Introduction to Hyaluronidases

The role and significance of hyaluronidases are perhaps best understood by starting with their substrate, hyaluronic acid. Originally elucidated by German biochemist Karl Meyer in 1934 [1], hyaluronic acid (HA) is a non-sulfated glycosaminoglycan (GAG) found in most vertebrates, and it has been largely associated with the pathogenesis of fibrosis [2]. While high-molecular-weight HA (HMW-HA) exerts anti-inflammatory and anti-fibrotic effects [3], low-molecular-weight HA (LMW-HA) promotes inflammation and fibrosis [4]. With this knowledge, the importance of hyaluronidases emerges as these enzymes are responsible for the cleavage of anti-fibrotic HMW-HA into pro-fibrotic LMW-HA.

Hyaluronidases (HYALs) themselves are endoglycosidases and are found in eukaryotes and prokaryotes alike. They were first observed by Duran-Reynals in 1928, who noted the rapid spread of bacteria, drugs, antiviral vaccines, and subcutaneously-injected India ink in an extract of mammalian testes. Appropriately, he coined the term “spreading factor” for this molecule [5], a property attributed to the fact that HYALs break down the structural integrity of HA, lowering its viscosity and leading to increased tissue permeability and thus spreading [6].

In 1971, the same Karl Meyer who detailed the chemical structure of HA classified the HYALs into three discrete classes of enzymes [7]. Meyers based his categorization of HYALs on their mechanism of action, with two groups comprised of endo-β-N-acetyl-hexosaminidases and one group of endo-β-glucuronidases. Of the former, one class of vertebrate enzymes leverages substrate hydrolysis [8], and the other makes use of bacterial eliminases, which employ β-elimination of the glycosidic linkage by way of an unsaturated bond [9]. The third group of HYALs, endo-β-glucuronidases, are primarily found in leeches [10], and although not much is known about this class of enzymes, their mechanism of action is thought to be more closely related to the vertebrate enzymes than their bacterial counterparts.

Critically, a large turnover of HA is common in vertebrate tissue [11]. In fact, levels of HA rise rapidly under pathological conditions, including burns, shock, and after major surgeries [12]. Understanding how HYALs promote HA degradation helps determine their influence in various disease states. As such, the next section of this review will specifically focus on HYALs in humans by detailing the HYALs and their mechanism of action.

## 2. Background and Mechanism of Action in Humans

**Figure 1:**
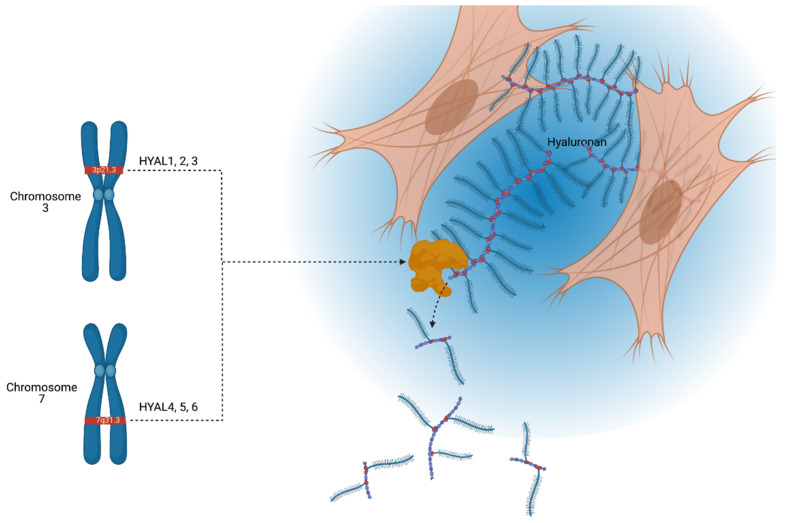
Schematic illustration depicting the chromosomal locations of hyaluronidases (*HYALs*) *1–6* and a secreted HYAL enzyme cleaving hyaluronic acid (HA). *HYALs 1–3* are located on chromosome loci 3p21.3, and *HYALs 4–6* on 7q31.3. Hyaluronidases are involved in the constant turnover of the extracellular proteoglycan hyaluronan, generating fragments of various molecular weights depending on the type of hyaluronidase. Pictured here is an extracellular hyaluronidase degrading HA, although it is important to note that HYAL may reside inside endosomes or be bound to the membrane surface. This figure is a broad illustration and is not drawn to scale. Created with BioRender.com.

Six hyaluronidase-like genes have been elucidated in the human genome, all of which are highly homologous, both within the paralog and across other vertebrates, including mice. In terms of location, *HYALs 1*, *2*, and *3* are found tightly clustered on chromosome loci 3p21.3, and *HYALs 4*, *5*, and *6* are found in a similar fashion on chromosome 7q31.3 [13]. *HYALs 5* and *6* are also known as *sperm adhesion molecule 1* (*SPAM1/PH20*) and *HYALP1*, respectively. *HYAL6* is known as *HYALP1* as it is a pseudogene that is transcribed but not translated in humans [14]. The arrangement of the genes suggests that one original gene sequence underwent two duplication events and a subsequent en masse duplication event to generate the chromosomal distribution found in humans and mice; notably, this means that properties of HYALs uncovered in murine studies can largely be translated to humans. We indicate the chromosomal locations of these six *HYALs* and their primary function, degradation of HA in the extracellular space, in Figure 1.

Among the six HYALs, HYALs 1 and 2 are the primary hyaluronidases responsible for the catabolism of HA in somatic tissue. Because *HYALs 3*, *4*, and *5* have weak expression patterns and *HYAL6* is a pseudogene, by process of elimination, these four HYALs likely do not participate in cleavage of HA [14]. (Refer to Table 1 for a summary of the human HYALs). However, this theory was properly corroborated by Csoka et al. when the group discovered that the mechanism for catabolism of HA involves HYAL2 working in lockstep with HA-receptor CD44 to break down HMW-HA into 20 kDa fragments. HYAL2, secured at the cell surface by glycosylphosphatidylinositol (GPI), produces the fragments, which are then packaged into acidic endosomes and transported intracellularly. Once inside the cell, β-glucuronidase, β-N-acetyl glucosaminidase, and HYAL1 further degrade the HA into LMW oligosaccharides [15]. Some modifications to this process may be required for certain specialized cell types, such as liver endothelial cells, in which HA-receptor for endocytosis (HARE) [16] and lymphatic vessel endothelial receptor 1 (LYVE-1) [17] may supplant the role of CD44.

Although HYALs 1–6 are the quintessential hyaluronidases, two other molecules, the hyaladherin KIAA1199 and the transmembrane protein TMEM2, also exhibit HYAL-like functionality in their degradation of HA. *KIAA1199* is a deafness gene with an elusive function that has been shown to depolymerize HA [18], and TMEM2, a cell surface molecule, has also been implicated in HA catabolism [19].

The studies presented in this section serve as a springboard for further research into the influence of HYALs in the development and progression of various human diseases. The remainder of this review will focus on the specific roles these enzymes play in multiple organ diseases and cancer. Refer to Table 2 for a complete summary.

## 3. Hyaluronidases in Human Organs and Cancer

### 3.1. In Skin

Of the known hyaluronidases, KIAA1199 likely has the largest role in skin, as it has been shown to be imperative to the ability of human skin fibroblasts to degrade HA [18]. KIAA1199 functions to degrade HA, which has been endocytosed by fibroblasts, with the metabolized products later excreted into the extracellular space [18]. Production of KIAA1199 in human fibroblasts increases in the setting of histamine, leading to an increase of HA degradation and suggesting a role of mast cells in the regulation of hyaluronan metabolism in human skin [20]. Histamine, however, has not been shown to affect TMEM2 expression in human skin fibroblasts, while TGF-ß1 does appear to induce TMEM2 [21]. The pro-inflammatory cytokines TNF-alpha, IL-1ß, and IL-6 appear to have the opposite effect, downregulating KIAA1199 in human fibroblasts and promoting HA synthesis via upregulation of HAS2 [22]. KIAA1199 has been implicated in the pathogenesis of facial wrinkles as the medicinal *Sanguisorba officinalis* root and *Geranium thungergii* extract have been shown to inhibit KIAA1199 and improve the appearance of facial wrinkles in small, randomly controlled trials [23,24]. Transgenic mice lacking HYALs 1 and 2 do not demonstrate a substantial accumulation of HA in the skin, with mice lacking HYAL1 accumulating HA primarily in joint cartilage while HYAL2-null mice have defects in the development of the axial skeleton and skull [25,26]. These findings in mice are at least partially corroborated in humans with a type of lysosomal storage disease termed mucopolysaccharidosis type IX (MPS IX), which results from a congenital deficiency of HYAL1. To date, this deficiency has only been reported in four patients, most of whom are members of a consanguineous family [27]. Patients with MPS IX typically demonstrate articular pathologies most resembling juvenile idiopathic arthritis, with no wound healing deficiencies reported in the literature [28].

In humans, the study of gene expression in tissue obtained from patients with keloid disease demonstrates reduced expression of *HYALs 1* and *2* when compared to normal skin [29]. Reduced serum HYAL1 activity has been shown in patients with advanced scleroderma, a systemic condition that results in dysregulated extracellular matrix (ECM) deposition with resultant pathologic fibrosis of even unwounded skin, as well as other organ systems [30]. A small cohort of patients with a spectrum of skeletal irregularities also had decreased levels of serum HYAL1 [31]. Averbeck et al. also examined the effect of ultraviolet radiation (UVB) on human skin expression of *HYAL*. They found that expression of *HYALs 1* and *2* is increased in human skin, specifically dermal fibroblasts, 24 h following exposure to UVB [32]. Thus, HYAL metabolism of dermal HA may play a role in the inflammation seen following a superficial sunburn. These limited studies in humans, when viewed in the context of the lens provided by animal studies, highlight the importance of the balance needed between HA synthesis and degradation in cutaneous response to injury. Pathologies may be associated with both an increase and decrease in HYAL expression, signifying the need for much further research into the role of hyaluronidases in wound repair.

Fetal fibroblasts demonstrate an HA-rich pericellular matrix (PCM) in vitro, a phenotype that may be important to the regenerative scarless wound healing seen in the mid-gestation fetus. An investigation into fetal fibroblasts demonstrates that the HA-rich PCM may result from a combination of increased *HAS* expression with a decrease in expression of *HYALs 1* and *2* and *KIAA1199* [33]. Clues from the involvement of HYAL in cutaneous wound healing primarily come from mouse studies. One such study demonstrated a decrease in expression of HYALs 1 and 2 in the wounds of older mice compared to young mice, with associated reduction of lower MW HA fragments as well as delayed wound closure in older mice [34]. These studies provide insight into the importance of hyaluronidase regulation in cutaneous wound healing, both in aspects of successful wound closure and involvement in regenerative tissue repair.

### 3.2. In the Cardiovascular System

A tight relationship between HA and HYAL is critical in the development and progression of heart disease, with different HYALs accounting for different phenotypes. In one study, researchers found that HYAL2 knockout (KO) mice accrued high extracellular HA levels, leading to cardiopulmonary dysfunction. This mouse model developed enlarged cardiac valves, a disorganized extracellular matrix (ECM), and severe lung fibrosis [35]. Another group echoed these findings when they demonstrated that human HYAL2 deficiency is associated with dilated coronary sinus, atrial enlargement, and a heart rare disease, cor triatriatum sinistrum [36,37]. In accordance with a second study, the same group suggested that an accumulation of HA stimulates endothelial-to-mesenchymal transition and subsequent proliferation of mesenchymal cells of HYAL2 KO mice, with the mice eventually developing heart failure [38]. This work confirms that HYAL2 could be a potential therapeutic target for the observed heart diseases in humans. In contrast, decreased *HYALs* were found to improve mouse heart tissue repair in another study. For background, our lab has shown that IL-10 is able to promote HA synthesis and reduce HA degradation in the skin and kidneys [33,35]. In this case, Jung et al. showed that reduced HYAL3 levels in a murine myocardial infarction (MI) model, mediated by IL-10, decreased HA degradation and subsequent collagen deposition. These results indicate that downregulation of *HYAL3* improves wound repair of the left ventricle post-MI by an IL-10-driven mechanism [39].

### 3.3. In the Lungs

Studies on hyaluronidases in human lung repair underscore their critical role in restoring damaged tissue. Congruent with the cardiovascular system, infected human lung fibroblasts showed that reduced HYAL2 expression contributes to elevated levels of HA and proteases, with subsequent binding to mast cells and exacerbated inflammation [40]. Another study validated this finding when vessel-derived HYAL2 promoted remodeling of the pulmonary vasculature and induced pulmonary hypertension (PH) in the absence of superoxide dismutase (SOD3), an enzyme thought to protect tissues from oxidative stress. The research suggests that inhibition of HYAL2 maintains homeostasis of HMW-HA, preventing proliferation by way of oxidation and hypoxia [41]. Another study showed that increased HYAL1 in a rat PH model could also exacerbate PH by digesting anti-inflammatory HMW-HA [42].

### 3.4. In the Kidneys

The function of hyaluronidases in kidney repair echoes studies of their role in the cardiopulmonary system. For instance, one study uncovered that shedding of HA, primarily mediated by HYAL1, contributes to the pathogenesis of various diseases, including ischemia/reperfusion (I/R), acute kidney injury (AKI), and chronic kidney disease (CKD). The research noted that elevated levels of HYAL are observed in dialysis patients [43] and that HYALs can serve as a viable biomarker and potential inhibition target to protect the HA composition of the endothelial glycocalyx (EG) [44]. Although the various HYALs are known for their enzymatic activity, non-enzymatic HYALs do exist, specifically a non-enzymatic HYAL2 that thrives in basic environments where it is separated from the CD44 receptor [45]. Even non-enzymatic HYAL2 can promote inflammation and fibrosis, as evidenced when such HYAL2 was found to mediate Ras homolog family member A (RhoA) and interact with the actin cytoskeleton, driving a pro-fibrotic phenotype in a rat model [46].

### 3.5. In the Liver

While the majority of HA is degraded locally in various tissues or in lymph nodes, a small amount escapes the lymph nodes in draining lymph and is introduced into systemic circulation, where it is metabolized and excreted predominantly by the liver and the kidneys [47,48,49,50]. The association between liver hyaluronan metabolism and injury is apparent due to the extensive study of the use of serum hyaluronan as a biomarker in both acute and chronic liver disease [50,51,52,53,54]. The elevated levels of serum HA in these disease states may indicate impaired HYAL activity in the liver. However, histochemical analysis of human liver samples has shown an increase in HYALs 1 and 2 in liver pathologies such as steatosis, steatohepatitis, and cirrhosis, indicating a more complex mechanism for increased serum HA with liver injury [55]. Serum hyaluronidase levels have also been shown to be elevated in patients with acute and chronic hepatitis C at the early stages of the diseases when compared to healthy controls [52]. TMEM2, however, was found to be decreased in patients with chronic hepatitis B infection compared to healthy controls [56]. In fact, TMEM2 may be protective against infection by hepatitis B, as shown in in vitro models using human liver cells, and may involve the JAK-STAT signaling pathway [57]. Multiple studies have shown that hepatic breakdown of HA occurs in rat and mouse models by sinusoidal endothelial cells and is principally executed by HYALs 1 and 2 [58,59]. Preclinical studies also show a similar trend of early serum hyaluronidase elevation in an animal model of toxic liver injury [51], and another study shows that HYAL2 is particularly involved in hepatic degradation of HA as sinusoidal endothelial cells of HYAL2 KO mice are laden with undigested HA [26].

These studies, in aggregate, indicate that a large knowledge gap exists with regard to the role of hyaluronidases in the injured liver. Further human studies are needed to determine the significance and possible therapeutic target of hyaluronidases in acute and chronic liver injury.

### 3.6. In the Gastrointestinal Tract

The limited research on HYALs in non-oncological GI studies reveals that elevated serum HYAL 1 is associated with the development of a pancreaticocutaneous fistula, a feared complication in which an abnormal epithelialized tract develops between the pancreatic duct and the skin in pancreatic cancer patients who underwent pancreaticoduodenectomy [60]. This finding indicates that serum measures of HYAL 1 can be used to predict the likelihood of developing a pancreaticocutaneous fistula. Additionally, this can aid in counseling and communicating risks to patients prior to undergoing pancreaticoduodenectomy or Whipple procedure. Another non-oncological GI disease process, inflammatory bowel disease (IBD), is associated with a reduction in HYAL2 in patients’ platelets, which has been shown to regulate trans-endothelial mononuclear cell migration in colonic tissue of IBD patients [61]. Patients who are suffering from Crohn’s disease, a type of inflammatory bowel disease, showed increased colonic fibroblast-specific KIAA1199, which is possibly induced by key inflammatory cytokine IL-6 [62].

Damage-associated molecular patterns (DAMPs) are a group of molecules induced by tissue damage that send signals to innate immunity and cause noninfectious inflammatory responses to allow the body to start repairing the damaged cells and tissues [63]. Elevated serum DAMP levels are associated with multiple human inflammatory diseases, including IBD and Crohn’s diseases [64]. Moreover, HA deposition and remodeling precede inflammation, suggesting that KIAA1199 could cleave HA into DAMPs in colitis mouse models. This may explain why IBD patients show increased DAMPs [65]. Another preclinical study has shown that failure of HA to bind to its receptor leads to reduced intestinal length and altered detrimental structure, demonstrating the importance of HA metabolism in intestinal development [66]. Hyaluronidases, however, are understudied in the intestinal tract, with research efforts focused on enteric inflammation, such as in Crohn’s disease.

### 3.7. In Cancer

HA metabolism is a critical factor in the tumorigenesis, invasion, and metastasis of cancer cells, invariably playing an important role in mortality. The accumulation of lower-molecular-weight hyaluronan as a result of HYAL in pathologic specimens from patients with colorectal cancer is associated with lymphatic invasion and lymph node metastasis [67]. KIAA1199 has been shown to be upregulated in colorectal cancer tissues, over-expression of which promotes cancer cell migration and invasion [68]. Patients with hepatocellular carcinoma samples that co-express KIAA1199 and hypoxia-inducible factor 1 alpha have elevated mortality compared to patients with low co-expression [69]. Additionally, KIAA1199 over-expression on its own is correlated with poor prognosis in gastric, lung, pancreatic, and colon cancers [70]. HYALs 1 and 2 are overexpressed in tumors of patients with prostate cancer, bladder cancer, and melanoma [71]. Contrarily, under-expression of HYALs 1 and 2 may lead to worse mortality in pancreatic, ovarian, and endometrial cancers, indicating that the maladaptive effects of hyaluronidases in cancer can vary depending on the specific tissue in which the cancer arises [71]. Hyaluronidases may serve as distinct targets for oncologic therapies as they can be tailored to the specific pathogenesis of a given cancer.

## 4. Hyaluronidase-Based Therapeutics

There are currently several FDA-approved drugs that leverage HYALs as a clinical therapeutic in the treatment of various conditions and diseases. This review will focus on two specific classes of HYAL-based medications, one that uses HYALs as a primary ingredient and another that uses HYALs as a secondary ingredient.

Amphadase, Hylenex, and Vitrase are three commonly prescribed medications that use HYALs as a primary active component. Each of these therapeutics uses HYALs as a subcutaneously-administered adjuvant to hydrolyze HA and thus increase the permeability of the connective tissue. As a result, injected medication or localized exudates or transudates are more readily absorbed [72,73,74]. Although they use the same mechanism, the HYALs in these drugs are derived from three different species: Amphadase uses a bovine testicular-derived HYAL, Hylenex uses a recombinant human HYAL, and Vitrase uses an ovine testicular-derived HYAL.

Darzalex Faspro, Herceptin Hylecta, HyQvia, and Rituxan Hycela comprise the next class of therapeutics that use HYALs as a secondary component to depolymerize HA in the subcutaneous ECM, similar to the first class of meds. The degradation of HA reduces tissue viscosity, thereby increasing the absorption of each respective primary active ingredient. This illustrates the versatility of HYALs as a supplementary therapeutic as each drug employs a different primary active ingredient to treat different diseases: Darzalex uses daratumumab to treat multiple myeloma, Herceptin uses trastuzumab to treat breast cancer, Hyqvia uses IgG as a replacement therapy for immunodeficiency, and Rituxan uses rituximab to treat various lymphomas [75,76,77,78]. All HYALs in this class are from a recombinant human source.

The drugs mentioned in this section are summarized in Table 3; these drugs, along with others currently undergoing clinical trials, showcase the therapeutic potential of HYALs and highlight the benefit of further translational research.

## 5. Concluding Thoughts

This review served to summarize the current studies on HYALs and, in turn, highlighted a gap in the primary research on intrinsic hyaluronan homeostasis in humans. Although much effort has been placed in investigating the pathologic role of hyaluronidase over- and under-expression in various cancers, little translational research has been performed on hyaluronidases as potential pharmacologic targets in non-oncologic disease processes. The pro-inflammatory LMW-HA product of HYAL metabolism has great impact on aesthetic dermatologic pathologies and is an area of ongoing investigation by many regenerative healing groups, including our own. The pro-fibrotic and pro-inflammatory effects of hyaluronidases are not unique to the skin. Targeting HYAL2 in the lung parenchyma and vasculature may aid in the treatment of pulmonary hypertension and pulmonary fibrosis, while anti-HYAL2 therapies may be of benefit to patients with progressive kidney disease. Patients with IBD may see benefit from a multimodal anti-inflammatory pharmacologic regimen that includes drugs against KIAA1199. We may one day include serum levels of hyaluronan in the Model For End-Stage Liver Disease (MELD) score used to stratify cirrhotic patients for liver transplant priority.

By investigating the role of HYALs in various diseases, further therapeutic benefits of the molecules can be uncovered. The encouraging studies presented in this review highlight the need for greater translational research on HYALs and their role in mediating HA MW. As future studies unveil more information about hyaluronidases, these findings may be leveraged to improve outcomes for patients across multiple organ diseases.

## Figures and Tables

**Table 1:** **Summary of the human hyaluronidases.** This table details the chromosomal location, coded protein, active pH, and somatic activity of each human hyaluronidase gene

Chromosomal Location	Gene	Protein	Active pH	Somatically Active
3p21.3	*HYAL1*	HYAL1	3–4	Yes
	*HYAL2*	HYAL2	4, 7.5	Yes
	*HYAL3*	HYAL3	/	No
7q31.3	*HYAL4*	HYAL4	/	No
	*HYAL5/SPAM1/PH20*	HPH-20	4, 7.5	Yes
	*HYAL6/HYALP1*	Noncoding	/	No
15q25.1	*CEMIP*	KIAA1199	/	Yes
9q21.13	*CEMIP2*	TMEM2	/	Yes

**Table 2:** Role of Hyaluronidases in various disease processes. This table details the known involvement of hyaluronidases in specific disease states.

Organ System	Disease/Condition	Hyaluronidase(s) Involved	Hyaluronidase Involvement
Skin and connective tissue	Mucopolysaccharidosis type IX (MPS IX)	HYAL1	Deficiency leads to joint pathology resembling juvenile idiopathic arthritis
	Keloid scarring	HYALs 1 & 2	Reduced expression compared to normal skin
	Scleroderma Facial wrinkles	HYAL1 KIAA1199	Reduced in serum Exacerbates facial wrinkles
Cardiovascular	Cor triatriatum sinistrum	HYAL2	Deficiency
	Post-myocardial infarction fibrosis	HYAL3	Increases collagen deposition
Pulmonary	Pulmonary hypertension	HYAL2	Promotes remodeling of vasculature and pulmonary hypertension
Renal	Chronic kidney disease	HYAL1	Elevated in serum of patients with end-stage renal disease
Hepatic	Steatosis, steatohepatitis, cirrhosis	HYALs 1 & 2	Elevated in diseased liver parenchyma
	Hepatitis B	TMEM2	Decreased in chronic infection; may be protective against infection
	Hepatitis C	HYALs	Elevated in serum
Gastrointestinal	Pancreaticocutaneous fistula	HYAL1	Elevated in serum
	Inflammatory bowel disease	HYAL2	Reduced in platelets
	Crohn’s disease	KIAA1199	Elevated in colonic fibroblasts
	Colorectal cancer	KIAA1199	Overexpression promotes cancer cell invasion

**Table 3:** List of approved drugs using HYALs. This table comprises a collection of drugs leveraging HYALs as either a primary or secondary component for the treatment of various conditions and diseases. All drugs are administered via subcutaneous injection, which benefits from HYALs degrading HA in the skin for increased absorption of the active ingredient. All listed drugs are approved by the FDA.

Brand Name	Composition	Mechanism	Use	Species Origin
Amphadase	HYAL	Dispersion agent to hydrolyze HA	Adjuvant for hydration	Bovine testicular
Darzalex Faspro	Daratumumab, HYAL	HYAL depolymerizes HA to increase absorption of daratumumab	Multiple myeloma	Recombinant human
Herceptin Hylecta	Trastuzumab, HYAL	HYAL depolymerizes HA to increase absorption of trastuzumab	Adjuvant for breast cancer	Recombinant human
Hylenex	HYAL	Dispersion agent to hydrolyze HA	Adjuvant for hydration	Recombinant human
Hyqvia	IgG, HYAL	HYAL depolymerizes HA to increase absorption of immunoglobulin G	Replacement therapy for immunodeficiency	Recombinant human
Rituxan Hycela	Rituximab, HYAL	HYAL depolymerizes HA to increase absorption of rituximab	Various lymphomas	Recombinant human
Vitrase	HYAL	Dispersion agent to hydrolyze HA	Adjuvant for hydration	Ovine testicular

## Abbreviations

DAMP	damage-associated molecular pattern
ECM	extracellular matrix
GAG	glycosaminoglycan
GPI	glycosylphosphatidylinositol
HA	hyaluronic acid/hyaluronan
HARE	HA-receptor for endocytosis
HMW-HA	high-molecular-weight hyaluronic acid
HYAL	hyaluronidase
IBD	inflammatory bowel disease
KO	knockout
LMW-HA	low-molecular-weight hyaluronic acid
LYVE-1	lymphatic vessel endothelial HA-receptor-1
MW	molecular weight
PH	pulmonary hypertension
